# Predicting HIV Status among Men Who Have Sex with Men in Bulawayo & Harare, Zimbabwe Using Bio-Behavioural Data, Recurrent Neural Networks, and Machine Learning Techniques

**DOI:** 10.3390/tropicalmed7090231

**Published:** 2022-09-05

**Authors:** Innocent Chingombe, Tafadzwa Dzinamarira, Diego Cuadros, Munyaradzi Paul Mapingure, Elliot Mbunge, Simbarashe Chaputsira, Roda Madziva, Panashe Chiurunge, Chesterfield Samba, Helena Herrera, Grant Murewanhema, Owen Mugurungi, Godfrey Musuka

**Affiliations:** 1 Graduate Business School, Chinhoyi University of Technology, Chinhoyi, Zimbabwe; 2 ICAP, Columbia University, Harare, Zimbabwe; 3 School of Health Systems & Public Health, University of Pretoria, Pretoria 0002, South Africa; 4 Department of Geography and Geographic Information Science, University of Cincinnati, Cincinnati, OH 45221, USA; 5 Department of Information Technology, Faculty of Accounting and Informatics, Durban University of Technology, Durban 4000, South Africa; 6 School of Sociology and Social Policy, University of Nottingham, Nottingham NG7 2RD, UK; 7 GALZ, Harare, Zimbabwe; 8 School of Pharmacy and Biomedical Sciences, University of Portsmouth, Portsmouth PO1 2UP, UK; 9 Unit of Obstetrics and Gynaecology, Department of Primary Health Care Sciences, Faculty of Medicine and Health Sciences, University of Zimbabwe, Harare, Zimbabwe; 10 Ministry of Health and Child Care, AIDS and TB Programme, Harare, Zimbabwe

**Keywords:** HIV/AIDS, status, MSM, deep learning, machine learning, prediction models

## Abstract

HIV and AIDS continue to be major public health concerns globally. Despite significant progress in addressing their impact on the general population and achieving epidemic control, there is a need to improve HIV testing, particularly among men who have sex with men (MSM). This study applied deep and machine learning algorithms such as recurrent neural networks (RNNs), the bagging classifier, gradient boosting classifier, support vector machines, and Naïve Bayes classifier to predict HIV status among MSM using the dataset from the Zimbabwe Ministry of Health and Child Care. RNNs performed better than the bagging classifier, gradient boosting classifier, support vector machines, and Gaussian Naïve Bayes classifier in predicting HIV status. RNNs recorded a high prediction accuracy of 0.98 as compared to the Gaussian Naïve Bayes classifier (0.84), bagging classifier (0.91), support vector machine (0.91), and gradient boosting classifier (0.91). In addition, RNNs achieved a high precision of 0.98 for predicting both HIV-positive and -negative cases, a recall of 1.00 for HIV-negative cases and 0.94 for HIV-positive cases, and an F1-score of 0.99 for HIV-negative cases and 0.96 for positive cases. HIV status prediction models can significantly improve early HIV screening and assist healthcare professionals in effectively providing healthcare services to the MSM community. The results show that integrating HIV status prediction models into clinical software systems can complement indicator condition-guided HIV testing strategies and identify individuals that may require healthcare services, particularly for hard-to-reach vulnerable populations like MSM. Future studies are necessary to optimize machine learning models further to integrate them into primary care. The significance of this manuscript is that it presents results from a study population where very little information is available in Zimbabwe due to the criminalization of MSM activities in the country. For this reason, MSM tends to be a hidden sector of the population, frequently harassed and arrested. In almost all communities in Zimbabwe, MSM issues have remained taboo, and stigma exists in all sectors of society.

## 1. Introduction

According to UNAIDS, in 2019, there were 36.2 million [30.2 million–42.5 million] adults and 1.8 million [1.3 million–2.2 million] children (0–14 years) living with HIV globally [1], up from 34 million in 2010 [2]. Although there has been remarkable progress in diagnosis and access to antiretroviral therapy (ART) [3], HIV prevention measures in sub-Saharan Africa are still short of attaining the UNAIDS 90–90-90 fast track targets set in 2014. Zimbabwe is one of the few countries in Africa to have made significant progress toward achieving HIV epidemic control, with findings from a recent national survey revealing that 86.8% of people living with HIV were aware of their status; among them, 97.0% were on ART and 90.3% of these were virally suppressed (ZIMPHIA Summary Sheet). As Zimbabwe has reached the UNAIDS 90-90-90 targets by 2020, focusing on men who have sex with men (MSM) will be crucial in ensuring the country can achieve and sustain the 95-95-95 UNAIDS targets before 2030 [4].

Notwithstanding the significant progress in HIV testing and treatment, there remains a need to develop innovative approaches to reach hidden population groups such as MSM. The challenges faced by MSM include criminalization, which tremendously hinders their access to HIV and other essential health care services in Zimbabwe. Despite substantial advances in the testing, prevention, and treatment of HIV/AIDS, the overall trend of HIV incidence among MSM has been consistently upwards. A recent study conducted by Zimbabwe’s Ministry of Health and Child Care (MoHCC) found that HIV prevalence among this hard-to-reach vulnerable community was higher than that of the general population (17.1% vs. 12.9%) [5]. The same study found that achieving the UNAIDS HIV testing targets is still meagre in MSM at about 44%. This poses a considerable threat to attaining HIV epidemic control for the country and the population. Furthermore, MSM are at higher risk of psychological distress than the general population due to health issues because of multiple factors, including stigmatization, discrimination, and isolation in the community [6].

Higher HIV/STIs, low HIV testing [7], and lack of engagement with innovative interventions such as digital interventions are common challenges among MSM [8]. Several interventions such as intelligent mobile applications [3], electronic mail, short message services [9], voice messages [6], social media platforms [10], and online virtual simulation intervention (socially optimized learning in virtual environments) have been utilised to alleviate impediments found in this hard-to-reach population. Some of these interventions can be implemented at a large scale with low costs to enhance health promotion, change risky behaviours [9], strengthen self-efficacy, and create awareness [11], and may be helpful to in the MSM community to improve the goal of early HIV screening. In the Western European Region, early universal HIV screening, low stigmatization, increased access to HIV testing, and sustained antiretroviral therapy are considered part of significant indicators for low HIV prevalence among MSM [12]. However, universal HIV screening is relatively expensive, especially in developing countries [13], as it involves securing testing kits. Therefore, there is a need to incorporate computational techniques that consider primary health data to identify when individuals should be prioritized for HIV screening [12]. Using their existing health data for this purpose may reduce the inequalities to which MSM are subject. This may also potentially contribute to identifying individuals at increased risk of acquiring HIV, including those who might be pre-exposure prophylaxis (PrEP) candidates. There is a need to devise predictive models for identifying individuals who are likely to test HIV positive within MSM communities to present conducive health facilities for its clinical diagnosis. Therefore, this study applied machine learning techniques to predict HIV status among MSM. These models can process large amounts of health data and infer useful patterns that healthcare professionals can utilise to improve healthcare service provision in the MSM community. Based on our knowledge, this study is one within the limited body of studies that applied deep learning and machine learning models in predicting HIV status among MSM specifically in developing countries in the sub-Saharan Africa region. The study sought to achieve the following objectives.

- Apply RNNs and machine learning models to predict HIV status among MSM.
- Compare the performance of RNNs and machine learning models in predicting HIV status among MSM.
- Propose recommendations for future research directions on applying deep learning and machine learning models in predicting HIV status among MSM.

The entire organization of the paper is based as follows: Section 2 presents the study methodology, Section 3 presents the study findings and discussion of these findings, and Section 4 presents the conclusion of the study based on study findings.

## 2. Methodology

### 2.1. Data Sources and Ethical Considerations

The study used secondary data collected as part of a prevalence study conducted by ICAP in 2018, targeting 1538 MSM from two prominent cities in Zimbabwe: Bulawayo and Harare. The protocol and tools used in this study were approved by the Columbia University Irving Medical Center Institutional Review Board (#IRB-AAAR8950), CDC ADS (#2018-444), and the Medical Research Council of Zimbabwe (#—MRCZ/A/2156). The protocol was also reviewed per the US Centers for Disease Control and Prevention (CDC) human research protection procedures. A bio-behavioural survey (BBS) collected demographic, behavioural, and bio-marker data on sexually transmitted infections (STIs), including Hepatitis B, syphilis, HIV status, and HIV Recency status. Interviews and health tests were conducted in private and secure spaces. Responses were captured using tablets that were programmed with the survey CTO Collect. Participation in the study was voluntary. Above all, informed consent, subjects’ privacy, and confidentiality of their data were also suitably observed during the work. The study used written informed consent. The survey had many components, and participants would consent to each component, for example, consent to be interviewed, consent for blood draw, consent to be tested for STIs, or consent to have blood stored for future studies. The participant would sign and date the consent form. No minors were included in this study.

HIV Recency data were available for all individuals who tested HIV positive and consented to be tested for HIV Recency. Tests for HIV status were used to determine if individuals were either positive or negative for HIV, whilst those for HIV Recency were conducted to determine if the HIV infection was recent (acquired less than 12 months from the date of the Recency test) or long-term (acquired more than 12 months before the Recency test).

The dataset included 863 features from the 1538 participants. These included all components extracted from the survey questionnaire: demographic, behavioural, and bio-marker data on STSs, including Hepatitis B, Syphilis, HIV status, and HIV Recency status. The dataset had no duplicates, meaning that the 1538 rows all represented different individuals. However, not all features from the dataset were relevant to predicting HIV status among MSM. We analysed several pieces of the literature that conducted studies in the same domain to determine significant predictors before applying feature selection models. Several studies alluded that income, awareness, and knowledge about HIV are essential HIV status predictors. Further, other predictors include a history of substance use, STIs, multiple male sex partners, specific sexual behaviours, and frequency of condom use [14]. In addition, demographic characteristics and health status indicators can predict HIV transmission risk among HIV-infected MSM [15].

A trial conducted by [16] considered predictors such as relationship status, self-reported history of the number of HIV/STI screens, STIs, and post-exposure prophylaxis (PEP) in the previous 12 months of sexual behaviour in the last 90 days as essential variables. Another study [17] applied machine learning models to predict the diagnosis of HIV and STIs using demographic, clinical, behavioural, and laboratory data from the clinic records of MSM. Their study posits that past syphilis infection, STIs symptoms, residential rurality, and frequency of condom use with casual male sexual partners during receptive anal sex in the past 12 months were the most critical predictors of HIV diagnosis [17]. A study conducted by [12] used demographic data, socio-economic characteristics, and STI history to predict HIV status. This study included different predictors to predict HIV status among MSM. The selected predictors (features) and their respective descriptions are shown in Table 1.

### 2.2. Data Preprocessing

**Figure 1:**
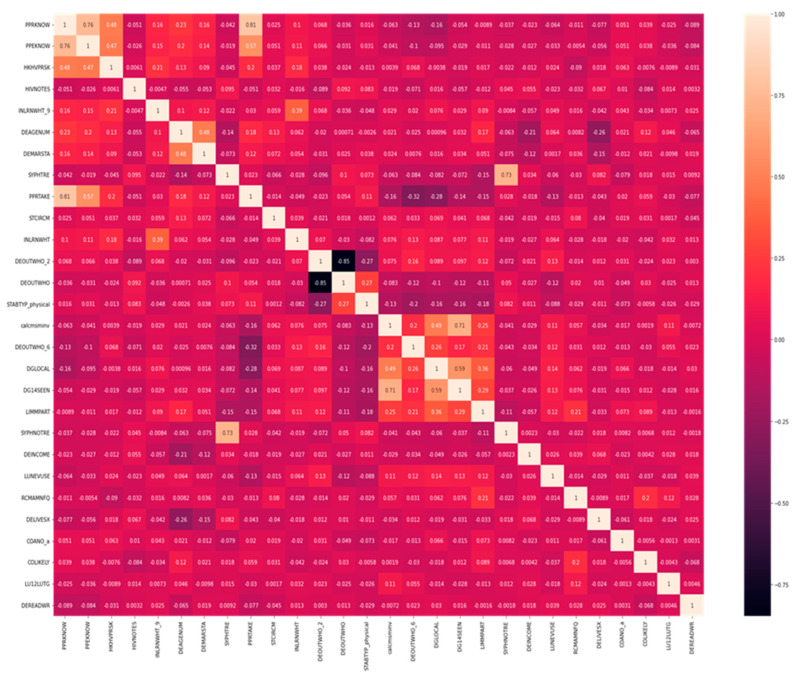
Correlation matrix of the selected features.

The dependent variable (hivresult) had three possible values, which were “negative”, “positive”, and “nan”. The “nan” values showed values that were missing values. The dataset had 27 “nan” values in the column for the HIV results, representing 1.76% of all the values in the queue. The null values in the HIV result column could indicate individuals who refused to consent to take an HIV test. The rows with null values were removed. Data preprocessing was performed computationally and objectively from the dataset with 863 features to select relevant components. Feature importance was performed using the Extra Trees classifier, also known as the Extremely Randomized Trees classifier, a variant of a random forest. The Extra Trees classifier is an ensemble learning technique that creates a group of unpruned decision trees following the traditional top-down method to output classification results [18]. The Extra Trees classifier randomizes attribute and cut-point selection when selecting essential features while splitting a tree node. The Extra Trees classifier differs from ensemble learning techniques; it splits nodes by picking cut-points at random. It also uses the whole training sample (instead of bootstrap replica) to grow the trees [19]. Independent features with high correlation were removed from selected features. Only 28 relevant features were selected using the Extra Trees classifier, and the heatmap correlation matrix of the selected features is shown in Figure 1.

### 2.3. HIV Status Prediction Models

**Figure 2:**
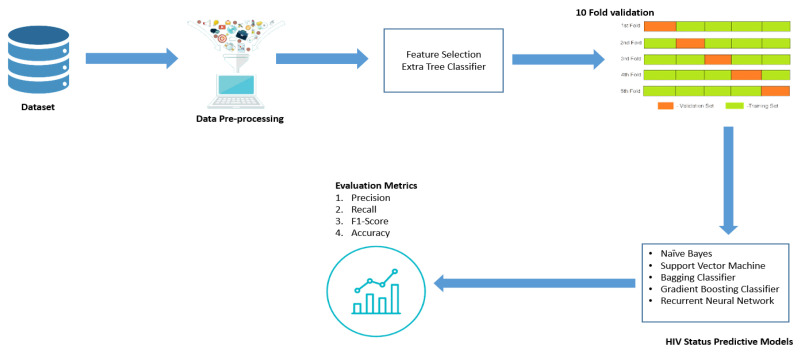
HIV status prediction models.

The proposed HIV status prediction model consists of input variables from the HIV dataset of MSM, data preprocessing, deep learning and machine learning models, and finally, the performance evaluation standard in three different phases. These phases are shown in Figure 2.

#### 2.3.1. Gaussian Naïve Bayes

The Gaussian Naïve Bayes classifier is a classification algorithm based on Bayes’ Theorem [20]. Naïve Bayes is not a single algorithm, but a family of algorithms that share a common principle, i.e., features being used to classify are assumed to be independent of each other. It predicts the membership probabilities for each class [12], and the class with the highest chance is considered the most likely [21]. Before deep learning, the Naive Bayes classifier was a commonly used classification algorithm. Apart from being simple, the Naïve Bayes classifier performs exceptionally well in many applications such as forecasting, classification recognition, and prediction.

#### 2.3.2. Support Vector Machines

The Support Vector Machine (SVM) is a supervised machine learning algorithm typically used to solve either classification or regression problems [20]. The SVM is a binary classification algorithm that classifies data and separates the two classes by constructing an operating separating hyperplane [22]. The support vectors are the data points closest to the hyperplane, while the hyperplane is a decision space divided between a set of objects with different classes [23]. All parameters for SVM in sklearn were left on default. We tried various combinations of parameters such as changing the C value, gamma value, and kernel type, but the defaults once produced the best results. We used gamma as auto, C as 1, and kernel as RBF.

#### 2.3.3. Bagging Classifier

A bagging classifier is an ensemble meta-estimator that fits base classifiers on random subsets of the original dataset and then aggregates their predictions (either by voting or averaging) to form a final prediction [24]. Such a meta-estimator can typically reduce the variance of a black-box estimator (e.g., a decision tree) by introducing randomization into its construction procedure and then making an ensemble out of it [25]. We set the bool parameter (bootstrap) to True to allow sampling with replacement. We also set n_jobs to 3 since we used a computer with three processors. We did this to reduce the time taken for execution.

#### 2.3.4. Gradient Boosting Classifier

Gradient boosting is a gradient-based approach to learning a boosting classifier incrementally [26]. Gradient boosting classifiers are a group of machine learning algorithms that combine many weak learning models to create a robust predictive model [27]. Decision trees are usually used when performing gradient boosting. The principle idea of the gradient boosting classifier is to construct the new base-learners to be maximally correlated with the negative gradient of the loss function associated with the whole ensemble [28]. The loss functions applied can be arbitrary, but to give a better intuition, the learning procedure would result in consecutive error-fitting if the error function is the classic squared-error loss [29]. To overcome overfitting, we reduced the learning rate from a default of 0.5 to 0.1, and tree depth to 6. Several hyperparameters were tuned to reduce overfitting and reduce loss. Several estimators were set to 10, and this number was experimental. We also put our learning rate at 0.5 to balance learning and prediction.

#### 2.3.5. Recurrent Neural Network

**Figure 3:**
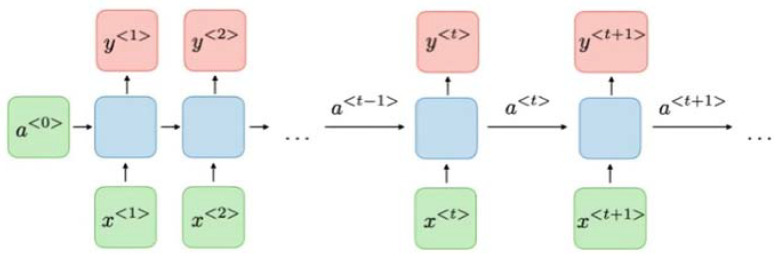
RNN Architecture.

Recurrent neural networks are deep neural networks that process sequential data (data in which order is important), such as audio processing or a series of words in a sentence [30,31]. This means RNNs can be used for NLP. RNNs allow previous outputs to be used as inputs while hiding the state [32]. RNNs apply the same operation on every sequence element, hence, the word recurrent. RNNs can be used, for instance, to predict the next word in a sentence [30]. Figure 3 shows the architectural design of RNNs.

The output $y^{<t>}$ and activation function $a^{<t>}$ for timestamp *t* are expressed as follows:

$$a^{<t>}=g1(W_{aa}a^{<t-1>}+W_{ax}x^{<t>}+b_{a}$$

$$y^{<t>}=g2\left(W_{ya}a^{<t>}+b_{y}\right)$$

where $W_{aa},\;W_{ax},\;W_{ya}$, $b_{a}$, and $b_{y}$ are temporarily shared coefficients, and $g_{1}$ and $g_{2}$ are activation functions. Wang et al. [33] adopted RNNs to forecast HIV incidence. For instance, RNNs have been widely used in clinical prediction tasks due to their strong modelling capacity in sequential data [34]. RNNs consume more memory during training and took more time compared to other algorithms used in the research. We used the loss as error measure to avoid overfitting.

### 2.4. Performance Evaluation Standards

The performance evaluation of the machine learning models was performed using precision, recall, accuracy, F1-score, and receiver operating characteristic (ROC) curve calculating the Area Under the Curve (AUC). The values of the evaluation metrics are calculated from the confusion matrix (CM). The matrix is composed of four categories. Firstly, true positives (TP) are examples correctly labelled as positives (MSM who are positive and classified as positive). Secondly, false positives (FP) refer to negative examples incorrectly labelled as positive (MSM who tested positive were classified as negative). Thirdly, true negatives (TN) (MSM who tested negative and were classified as negative) correspond to negatives correctly labelled as negative. Lastly, false negatives (FN) refer to positive examples incorrectly labelled as negative. We can use these categories to determine each model’s precision, recall, accuracy, F1-score, and AUC. Precision is the number of true positives separated by the number of false positives and true positives [35]. It is calculated as follows:

$$\mathrm{Precision}=\frac{\mathrm{TP}}{\mathrm{TP}+\mathrm{FP}}$$

A recall is the number of true positives to all positive class instances. It is calculated as follows:

$$\mathrm{Recall}=\frac{\mathrm{TP}}{\left(\mathrm{TP}+\mathrm{FN}\right)}$$

Accuracy is the percentage of correctly classified positive and negative examples [36]. It is calculated as follows:

$$\mathrm{Accuracy}=\frac{\mathrm{TP}+\mathrm{TN}}{\left(\mathrm{TP}+\mathrm{TN}+\mathrm{FP}+\mathrm{FN}\right)}$$

The F1-score is the balance between recall and precision [37]. It is calculated as follows:

$$F1-\mathrm{Score}=2\times \;\frac{\mathrm{Precision}\;\times \;\mathrm{Recall}}{\left(\mathrm{Precision}+\mathrm{Recall}\right)}$$

A receiver operating characteristic (ROC) curve is a graph showing the performance of a classification model at all classification thresholds. This curve plots two parameters namely, True Positive Rate (TPR) and False Positive Rate (FPR). TPR is a synonym for recall [38]. ROC is created by using a recall plot against a false positive rate (1-specificity) at different threshold values. The Area Under the Curve is another helpful measure in performance measures [39]. The AUC of ROC is a discrimination measure that tells us how well our predictor can classify MSM into the following two groups: those with HIV and those without HIV. AUC stands for “Area under the ROC Curve.” That is, the AUC measures the entire two-dimensional area underneath the entire ROC curve [40]. The AUC provides an aggregate measure of performance across all possible classification thresholds.

## 3. Results

We used 10-fold validation and the results are presented in Table 2. The results in Table 2 show that the Gaussian Naïve Bayes algorithm had a precision rate of 0.92 for predicting HIV-negative results, and 0.61 for predicting HIV-positive results. The model had a better recall rate for HIV negatives at 0.87 than the recall for HIV positive, which stood at 0.71. In terms of the F1-score, the Gaussian Naïve Bayes algorithm recorded the lowest score for the HIV-negative instances at 0.90, compared to 0.65 for the prediction of HIV-positive instances.

**Figure 4:**
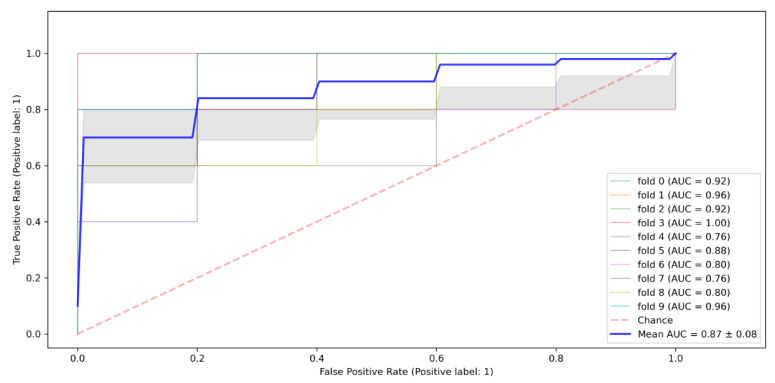
Naïve Bayes ROC algorithm ROC Curve.

Overall, the results from the Gaussian Naïve Bayes algorithm reveal that the model had an accuracy rate of 0.84, meaning that it could correctly predict true HIV positives as positive and true negatives as negatives for 0.84 of the instances/cases in the test dataset. Regarding the model’s performance as measured by the ROC curve’s Area Under the Curve (AUC), the Gaussian Naïve Bayes algorithm scored 0.87, as shown in Figure 4.

**Figure 5:**
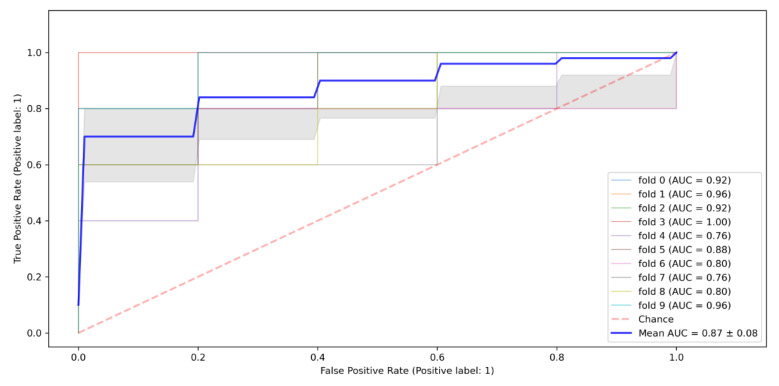
Support Vector Machine’s ROC Curve.

The SVM evaluation metrics revealed that the model had a higher precision score for predicting the HIV-positives instances at 0.97, compared to other machine learning models. Still, the HIV-negative scores’ prediction stood at 0.90. In terms of the model’s recall, which measures the model’s sensitivity, the results showed that the model performed better for the recall for the HIV-negative instances at 0.99 compared to the recall for the HIV-positive instances, which was 0.59. The SVM model’s F1-score was higher for the HIV-negative instances at 0.94 than for HIV-positive ones, which stood at 0.73. The overall accuracy for the SVM model was 0.91. Regarding the model’s performance as measured by its AUC for the ROC curve, the model had a score of 0.82, as shown in Figure 5.

**Figure 6:**
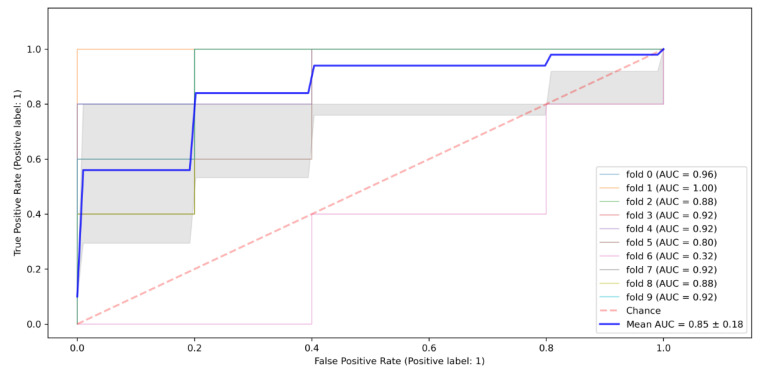
Bagging classifier ROC Curve.

The bagging classifier recorded a higher precision score for predicting HIV-negative instances, at 0.91, than its precision score for HIV positive, which stood at 0.89. Regarding the model’s recall scores, the model had a better score for the HIV-negative instances at 0.98, compared to the 0.64 for its recall for the HIV-positive instances. The model’s F1-score for the HIV-positive and -negative instances was also higher (0.94) than for the HIV positive. The model’s overall accuracy rate stood at 0.91. As assessed by the AUC of its ROC curve below, its performance was 0.87, as shown in Figure 6.

**Figure 7:**
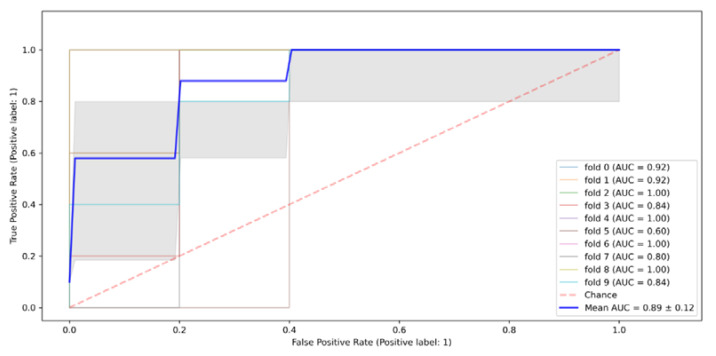
Gradient boosting algorithm ROC curve.

The gradient boosting algorithm’s performance results show that its precision for the prediction of the HIV results was higher for the HIV-negative instances at 0.91, compared to that for the prediction of HIV-positive results, which stood at 0.89. However, the model’s recall capacity was higher for the HIV-negative instances, at 0.94 compared to 0.64 for the recall of HIV-positive instances. Its F1-score was higher for the HIV-negative instances, at 0.94, than for the HIV-positive instances, which was pegged at 0.74. The model’s overall accuracy rate was 0.91. In terms of the model’s performance as measured by the ROC curve’s area under the curve, the algorithm scored 0.91, as shown in Figure 7.

**Figure 8:**
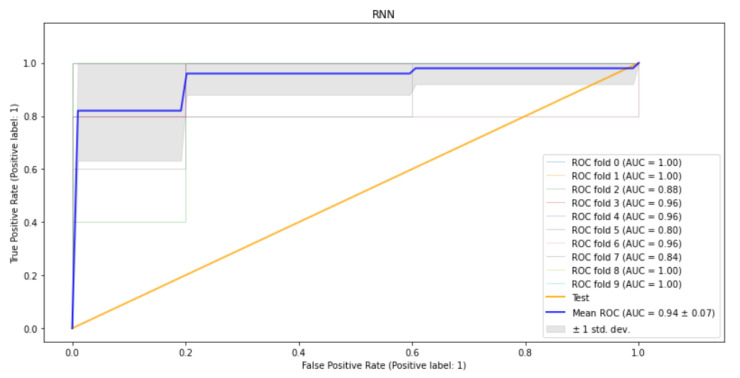
RNN’s training and testing loss.

The RNN model’s performance results show that the model’s precision for the HIV results was higher for the HIV-negative instances at 0.99, compared to the prediction of HIV-positive results, which stood at 0.92. However, the model’s recall capacity was higher for the HIV-negative instances, at 1.00, compared to 0.98 for the recall of HIV-positive instances. Its F1-score was higher for the HIV-negative instances, at 0.99, than for the HIV-positive instances, which was pegged at 0.96. The model’s overall accuracy rate was 0.99. The algorithm reached optimum accuracy after 12 epochs using the Early Stopping method to avoid overfitting as shown in Figure 8.

### 3.1. Policy Recommendations on the Application of Deep Learning and Machine Learning in Predicting HIV among MSM

Before key policy recommendations can be drawn from this work, there is a need to validate the model by enrolling members of the MSM community for classification of their HIV status before the same individuals are given an actual HIV test diagnosis, for which the results from the two will be compared for accuracy. Once the desired level of model classification performance is reached, the tool can be presented to the Zimbabwe Ministry of Health and Child Care for consideration as a pre-test HIV screening tool for members of the MSM community or for modification to include data for the general population. Tools such as a prediction model will assist HIV programmes in Zimbabwe and southern Africa to better address the needs of MSM. After that, it is essential to include this model and its attributes in health ministry policies and guidelines in order to ensure that the model is fully utilised.

### 3.2. Limitations

This study provides insights into some factors predicting HIV status among men who have sex with men using recurrent neural networks and machine learning techniques. Its findings need to be considered in public health policy, strategy, and practice. However, the cross-sectional design sampling technique and resultant distribution of demographic characteristics may have prevented all relevant factors associated with predicting HIV status among men who have sex with men from being identified. Cross-sectional studies do not show a temporal relationship between exposures and outcomes as longitudinal studies would. In addition, it may also have been underpowered to detect differences in some variables. Therefore, more extensive studies with a bigger sample size to accord higher statistical power may be required to explore all potential variables of interest fully.

All questionnaire data (condom use, history of HIV testing, awareness of status, and ART use) were self-reported and may be subject to social desirability bias. Although interviewers were trained in techniques to put participants at ease and support the accurate reporting of dates and events, self-reported data remain susceptible to recall bias and social desirability bias concerning sensitive topics, especially considering that same-sex sexual behaviours are illegal and highly stigmatized in Zimbabwe. Those that were recruited and agreed to participate may be a self-selected group of individuals more comfortable disclosing their sexual behaviour. The survey was limited to Harare and Bulawayo and did not reflect MSM and TGW/GQ activity throughout all of Zimbabwe.

## 4. Conclusions

Recurrent neural networks, bagging, gradient boosting, the support vector machine, and Gaussian Naïve Bayes classifier were applied to predict HIV status among MSM. In circumstances like this, where stigma and fear of prosecution prevent access to services, methods such as recurrent neural networks, the bagging classifier, gradient boosting classifier, support vector machine, and Gaussian Naïve Bayes classifier can successfully provide essential information on MSM, and potentially other hard-to-reach groups. HIV prediction models applied in this study used the known HIV risk factors of individuals exposed to different risks and at varying degrees of exposure. This study revealed that machine learning classifiers could significantly improve HIV testing capacity among MSM, and their use should be advocated. Recurrent neural networks achieved a high prediction accuracy of 0.98 as compared to other machine learning models. In addition, RNNs achieved a high precision of 0.98 for both HIV-positive and -negative cases, a recall of 1.00 for HIV-negative cases and 0.94 for HIV-positive cases, and an F1-score of 0.99 for HIV-negative cases and 0.96 for positive cases. Integrating HIV status prediction models into clinical software systems may complement existing strategies, such as indicator condition-guided HIV testing, and help identify individuals that may require healthcare services among the MSM community. With continued HIV research, including research targeting MSM in Zimbabwe, further knowledge on HIV among this group is likely to become more readily available, thereby increasing the amount of data to be used in the training of HIV predictive models and ultimately resulting in the improvement of the model performance beyond the current study’s best accuracy of 0.93. Establishing HIV status predictive models is essential for intensifying HIV testing and providing healthcare services, especially to the MSM community. However, future studies are required to optimize deep learning and machine learning models further and integrate them into primary care settings by incorporating HIV risk factors, clinical data, and socio-behavioural-driven data. This research helped identify the key questions that help in predicting HIV status among the MSM community.

Additionally, methods such as those used in this manuscript are increasingly used in studies addressing various issues in the health sector [41,42]. Future research will explore how these mentioned studies and others can provide additional ideas to enhance our studies. Our work in the future will use recurrent neural networks and machine learning techniques to understand factors associated with improved adherence to antiretroviral treatment and pre-exposure prophylaxis by MSM in the country.

## Figures and Tables

**Table 1:** Description of features.

Feature Name	Feature Description
PPRKNOW	Knowledge of Pre-exposure prophylaxis as (PrEP)
PPEKNOW	Knowledge of Post-exposure prophylaxis as (PEP)
HKHVPRSK	Self-perceived chances of becoming HIV infected in the next 12 months
DEAGENUM	Age in completed years
HIVNOTES	HIV Services where one was ever referred to
DEMARSTA	Marital status
INLRNWHT_9	Desire to learn more HIV treatment
SYPHTRE	Syphilis test result
INLRNWHT	HIV-related topics to learn more about
PPRTAKE	Ever taken PrEP
STCIRCM	Circumcision status
DEOUTWHO_2	Disclosed sexual identity to family members
DEOUTWHO_6	Disclosed sexual identity to a Health care provider
LUFREE	Ever been given “packets” of lubricant for free? For example, through an outreach service, drop-in centre, or health clinic, in the last six months
INLRNWHT_2	Desire to learn more about how to prevent HIV
LUTYPE_c	Use of water-based lubricant (Durex, etc.) during anal sex in the last 6 months
RCFEMNA	Type of sex (anal, oral, both), during last sex with main female partner
INLRNWHT_1	Desire to learn more about HIV prevention
DEATTRA	Sex/gender most sexually attracted to
LUNEVUSE	The main reason for not using a lubricant during anal sex in the past six months
DELIVESX	Currently living with a sexual partner or not
DEINCOME	Last monthly income
COANO_a	Condom use during anal sex when drunk
RCMAMNFQ	Frequency of condom use with the male partner one has sex with the most, in the last 6 months
DEREADWR	Ability to read and write
COLIKELY	Whether one is likely to use the condom when a man inserts his penis into his anus (butt) or when he is the one inserting a penis into someone’s anus or equally likely for both cases
LU12LUTG	Frequency of use of lubricants during anal sex with a man or transgender woman, in the last six months

**Table 2:** Performance of HIV status prediction models.

Prediction Model	Precision		Recall		F1-Score		Accuracy	AUC
	Negative	Positive	Negative	Positive	Negative	Positive		
RNN	0.98	0.98	1.00	0.94	0.99	0.96	0.98	0.94
Gaussian Naïve Bayes	0.89	0.65	0.88	0.68	0.88	0.66	0.83	0.87
Bagging Classifier	0.89	0.90	0.96	0.62	0.92	0.73	0.90	0.85
SVM	0.89	0.96	0.93	0.62	0.91	0.75	0.91	0.81
Gradient Boosting Classifier	0.91	0.89	0.97	0.65	0.94	0.75	0.91	0.89

## Data Availability

The data used in this study is available upon reasonable request from the Ministry of Health and Child Care, Zimbabwe.
